# Internal mammary artery injury during percutaneous coronary intervention: a case report

**DOI:** 10.1186/s12872-018-0972-4

**Published:** 2018-12-04

**Authors:** Zhicong Zeng, Yaqin Chen, Yinzhi Song, Fengxia Lin

**Affiliations:** 1 Cardiology Department, Affiliated Baoan TCM Hospital, Guangzhou University of Traditional Chinese Medicine, No.25, Yuan Second Road, Shenzhen City, Guangdong Province People’s Republic of China; 2 Cardiology Department, The Second Xiangya Hospital of Central South University, Changsha, China

**Keywords:** Complications, diagnostic catheterization, Percutaneous coronary intervention (PCI), Vascular, closure, Bleeding

## Abstract

**Background:**

Percutaneous coronary intervention (PCI) is widely used to treat coronary artery disease (CAD). However, complications of PCI are inevitable. Internal mammary artery (IMA) injury is an infrequent but potentially lethal complication of PCI.

**Case presentation:**

A 78-year-old man was diagnosed with multivessel lesions by coronary angiography. The IMA was injured during PCI, then cured by early identification and active rescue.

**Conclusions:**

This is the first reported case, to our knowledge, of injury to the IMA during PCI. We we report this case to discuss how to treat this injury effectively and avoid this complication during clinical therapy.

## Background

Percutaneous coronary intervention (PCI) can improve coronary flow and relieve the symptoms of myocardial ischemia, and is therefore widely used to treat coronary artery disease (CAD) [1]. However, it is an invasive treatment, and complications are inevitable. Common complications include coronary dissection, ano-reflow of coronary artery, stent thrombosis, perforation and cardiac tamponade, thrombus, puncture site hematoma, pseudoaneurysm, arteriovenous fistula, ventricular fibrillation, and contrast-induced nephropathy [2]. Immediate recognition of complications and prompt treatment is vital. Here, we report a case of injury to the internal mammary artery (IMA)—an infrequent but potentially lethal complication during PCI—that was repaired using a general vascular closure device.

## Case presentation

**Fig. 1:**
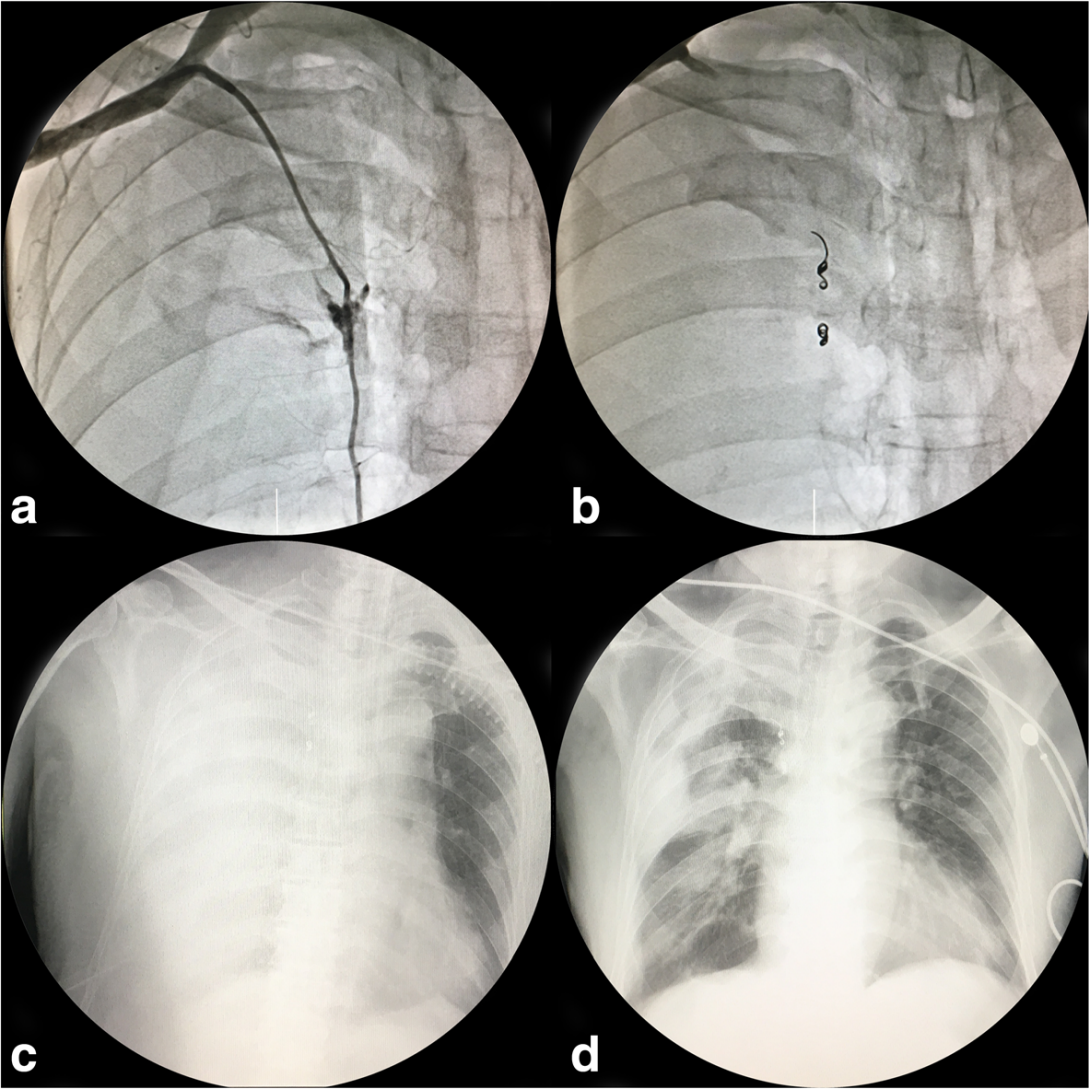
IMA angiography shows exudation in the third rib segment of the right IMA (**a**) The hemorrhage was staunched by embolization with coils (**b**). Chest radiograph shows a large shadow in the right chest suggestive of a pleural haemothorax (**c**). The pleural haemothorax is significantly reduced after treatment (**d**)

A 78-year-old man with CAD risk factors of diabetes mellitus, smoking, and hypertension was transferred to our department from a local hospital with the diagnosis of unstable angina pectoris. The percutaneous coronary intervention was performed by an attending physician with 3 years of interventional experience, and was guided by an expert operator. Coronary angiography performed via the right radial artery revealed 70% occlusion of the proximal segment of the left main anterior coronary artery (LM) and 90% occlusion of the proximal segment of the left anterior descending coronary artery (LAD). The diagnostic catheter(6F TIG, TERUMO, Japan) was withdrawn, and the hydrophilic guidewire (Merit Laureate; Merit Medical, USA) was advanced. Unnoticed, it strayed into the distal right IMA. When the guiding catheter (6F EBU3.5, Medtronic, USA), advanced over the guidewire, reached the proximal-middle segment of the IMA, the patient complained of intolerable chest pain. The guiding catheter and guidewire were immediately withdrawn. The guidewire was reintroduced into the aortic sinus and the guiding catheter was delivered to the left coronary artery, and balloon dilatation and stenting of the LM and LAD was performed. The patient again complained of severe chest pain, and his blood pressure began to fall. His condition deteriorated despite administration of opioid analgesics and intravenous fluids (Fig. 1a). Transthoracic echocardiography ruled out cardiac tamponade and aortic dissection. Fluoroscopy was suggestive of a right-sided pleural haemothorax (Fig. 1c). IMA angiography revealed obvious exudation of contrast in the third rib segment of the right IMA. A 2.0 mm × 15 mm semi-compliant balloon (MINI TREK, Abbott, IL, USA) was introduced up to the site of the leak and kept inflated for 20 min to reduce the exudation. Bleeding was finally staunched by embolization with coils (Fig. 1b). However, the patient developed cardiac shock and suffered a cardiac arrest. He was successfully resuscitated and transferred to the cardiac care unit, where he was intubated and mechanically ventilated (Fig. 1d). He was treated with chest tube drainage, intravenous fluids, and blood transfusion. Subsequently, after cardiac rehabilitation, he was discharged from hospital 23 days after admission.

## Discussion and conclusions

This case report describes a rare but potentially lethal complication of PCI.

The IMA arises from the first segment of the subclavian artery and descends upon the parietal pleura in the upper intercostal spaces [3]. The artery is often injured in patients who suffer sternal fracture or penetrating parasternal injury. IMA injury is an uncommon complication of central venous catheter placement [4, 5]. Rarely, it can be a life-threatening complication of PCI and other endovascular procedures. The complication can be avoided by ensuring correct placement of the guidewire before delivering the guiding catheter. Furthermore, the left anterior oblique view and right anterior oblique view are not the most appropriate for judging the position of the guidewire as there is some overlap of the IMA and the aorta in these views; an anteroposterior projection displays the position more reliably and may help avoid this complication.

Flow rates in the IMA average 150 mL/min, and massive hemorrhage can result from injury to the artery [6]. The possibility of iatrogenic injury to the IMA should be considered in any catastrophic situation during PCI. Emergency blood transfusion and fluid infusion can maintain the patient’s vital signs, and prompt chest tube drainage can help re-expand the lung. Selective arterial embolization is an option for emergency treatment of IMA injury. The choices for embolization material are microcoil [7], spongel [8] and covered stent grafts [9]. However, IMA embolization would result in loss of the ideal bypass conduit for coronary artery bypass grafting, and the decision must therefore be made after careful consideration of the pros and cons [10].

IMA injury is a very rare complication of PCI, which may cause hemothorax, severe shock, or even death. Before delivering the guiding catheter, the position of the guidewire should be confirmed to avoid this complication. Prompt diagnosis, effective embolotherapy, adequate drainage, and aggressive resuscitation are recommended for patients with IMA injury during PCI.

## Abbreviations

CAD: Coronary artery disease
IMA: Internal mammary artery
LAD: Left anterior descending coronary artery
LM: Left main anterior coronary artery
PCI: Percutaneous coronary intervention
